# Two Points in Administering Anæsthetics

**Published:** 1898-03-19

**Authors:** 


### TWO POINTS IN ADMINISTERING ANÆSTHETICS.

In a clinical lecture delivered at the London Hospital, Dr. Hewitt gives two warnings, one about ether and the other about chloroform, which may well be borne in mind. First about ether, it is pointed out that, both in going under and in coming out, there is a danger of self-asphyxiation, partly from excessive formation of mucus and partly from the fact that the acts of swallowing, which are apt to be set up at this period, are performed very tardily. During normal deglutition the glottis closes momentarily, but during the passage into profound anæsthesia the act of deglutition may be spread out, so to speak, over a considerable time, during which no air enters or leaves the chest. In the vast majority of cases this impaired breathing, which comes on just before stertor, passes off spontaneously, or may be made to do so by rubbing the lips briskly with a towel and pushing the lower jaw forward; but it may be necessary to separate the teeth and pass the finger to the back of the pharynx, when breathing will recommence. Much the same condition may arise during returning consciousness, and it is an excellent plan in all cases in which such a course is possible to turn the patient well upon the side immediately the anæsthetic is discontinued. Patients should be very carefully watched while they are emerging from deep anæsthesia, and tracheotomy instruments should be at hand.

The warning about chloroform is in regard to children. Small children are said to take chloroform particularly well, and this is no doubt true; but when once they have been brought under its influence they are very easily overdosed. It is often difficult to obtain anæsthesia, because the vapour readily causes the glottis to close, so that some time elapses before sufficient chloroform enters the lungs; but when once that stage is passed the anæsthetic will be absorbed freely, so that very small quantities are needed.

